# Magnesium Supplementation Alleviates the Toxic Effects of Silica Nanoparticles on the Kidneys, Liver, and Adrenal Glands in Rats

**DOI:** 10.3390/toxics11040381

**Published:** 2023-04-17

**Authors:** Mohamed Moharram Badawy, Mohamed Z. Sayed-Ahmed, Yosif Almoshari, Saad S. Alqahtani, Saeed Alshahrani, Heba Allah Ali Mabrouk, Marwa M. Abd-Elsalam, Khalid Alkashif, Sarfaraz Ahmad, Ahmed M. El-Sebaey, Mohamed G. Hamama, Dalia Alsaied Moustafa Ahmed

**Affiliations:** 1 Forensic Medicine and Clinical Toxicology Department, Faculty of Medicine, Mansoura University, Mansoura 35516, Egypt; 2 Forensic Medicine and Clinical Toxicology Department, Faculty of Medicine, Delta University for Science and Technology, Gamasa 11152, Egypt; 3 Department of Clinical Pharmacy, College of Pharmacy, Jazan University, Jizan 45142, Saudi Arabia; 4 Department of Internal Medicine and Infectious Diseases, Faculty of Veterinary Medicine, Mansoura University, Mansoura 35516, Egypt; 5 Department of Pharmaceutics, College of Pharmacy, Jazan University, Jizan 45142, Saudi Arabia; 6 Department of Pharmacology and Toxicology, College of Pharmacy, Jazan University, Jizan 45142, Saudi Arabia; 7 Forensic Medicine and Clinical Toxicology Department, Faculty of Medicine, Kafrelsheikh University, Kafr el-Sheikh 33516, Egypt; 8 Department of Histology, Faculty of Medicine, Kafrelsheikh University, Kafr el-Sheikh 33516, Egypt; 9 Department of Clinical Pathology, Faculty of Veterinary Medicine, Mansoura University, Mansoura 35516, Egypt; 10 Anatomy Department, Faculty of Medicine, Tanta University, Tanta 31527, Egypt

**Keywords:** silica nanoparticles, oxidative stress, liver, kidney, adrenal gland, magnesium

## Abstract

Concerns regarding the possible hazards to human health have been raised by the growing usage of silica nanoparticles (SiNPs) in a variety of applications, including industrial, agricultural, and medical applications. This in vivo subchronic study was conducted to assess the following: (1) the toxicity of orally administered SiNPs on the liver, kidneys, and adrenal glands; (2) the relationship between SiNPs exposure and oxidative stress; and (3) the role of magnesium in mitigating these toxic effects. A total of 24 Sprague Dawley male adult rats were divided equally into four groups, as follows: control group, magnesium (Mg) group (50 mg/kg/d), SiNPs group (100 mg/kg/d), and SiNPs+ Mg group. Rats were treated with SiNPs by oral gavage for 90 days. The liver transaminases, serum creatinine, and cortisol levels were evaluated. The tissue malondialdehyde (MDA) and reduced glutathione (GSH) levels were measured. Additionally, the weight of the organs and the histopathological changes were examined. Our results demonstrated that SiNPs exposure caused increased weight in the kidneys and adrenal glands. Exposure to SiNPs was also associated with significant alterations in liver transaminases, serum creatinine, cortisol, MDA, and GSH. Additionally, histopathological changes were significantly reported in the liver, kidneys, and adrenal glands of SiNPs-treated rats. Notably, when we compared the control group with the treated groups with SiNPs and Mg, the results revealed that magnesium could mitigate SiNPs-induced biochemical and histopathologic changes, confirming its effective role as an antioxidant that reduced the accumulation of SiNPs in tissues, and that it returns the levels of liver transaminases, serum creatinine, cortisol, MDA, and GSH to almost normal values.

## 1. Introduction

The design and manufacture of numerous types of nanoparticles (NPs) are encouraged by the growing application of nanomaterials in nearly every field of science, including chemistry, physics, materials science, molecular biology, reproduction, biotechnology, and engineering [1]. Moreover, NPs are utilized in the medical industry for drug delivery, imaging, and diagnosis [2]. There are increasing questions about the potential harm that nanoparticles could do to human and animal health as their usage has increased. As NPs have a greater surface area to volume ratio than larger particles, they have a greater impact on the environment and interact with other materials more strongly [3].

A common nanomaterial is silica nanoparticles (SiNPs), which are inorganic engineered materials that range in size from 1 to 100 nm. They possess unique characteristics including a large specific surface area, easy production and amplification, and facile surface modification. Over the last decade, silica nanoparticles have brought important innovations to many industrial and consumer sectors [4].

Silica nanoparticles are utilized in hundreds of products available on supermarket shelves, such as cosmetics, and personal care products, as well as some food products such as cheese products, fat and oil emulsions, vegetable oils, salt substitutes, dairy analogues, sugars and syrups, cereal-based foods, processed potato products, meat preparations, herbs and spices, soups, noodles, coffee creamer, and flavored drinks [5,6,7]. In addition, SiNPs have been widely developed for biological applications, including cancer treatment, DNA or drug delivery, biomarkers, and biosensors [8].

As human exposure to the SiNPs is increasing, the assessment of the toxicity of these nanoparticles is urgently needed. Exposure to SiNPs has evident toxicological consequences on biological systems. The toxic effects of SiNPs can be related to their size, which causes an exponential increase in the surface area. In addition, the shape plays an important role in toxicity, particularly when interacting with the cells [9]. Many toxic mechanisms of SiNP-induced organ toxicities have been described. These include penetrating the nucleus, causing DNA damage as well as the accumulation of intra-nuclear proteins in the cells, oxidative damage, pro-inflammatory response, genotoxicity, and apoptosis [10].

In vivo toxicology studies have suggested that SiNPs can induce adverse effects in different body organs. In male Wistar rats that were exposed orally to 10–15 nm SiNPs, histopathological examinations revealed gross tissue damage in kidney (swelling and necrosis of cells), lung (interstitial pneumonitis and bronchopneumonia), and in the testis (impaired spermatogenesis). Furthermore, blood biochemical parameters such as albumin, alkaline phosphatase, and aspartate aminotransferase levels have shown significant increases indicating liver dysfunction [11]. Similarly, Nemmar et al. [12] reported that exposure to SiNPs was associated with oxidative stress, inflammation, and DNA damage in the lung, liver, kidneys, and brain. In their study, SiNPs could react with oxygen molecules and produce superoxide and other reactive oxygen species (ROS), causing oxidative damage.

Other studies have also assessed the harmful effects of SiNPs (ranging in size from 14 to 30 nm) on various cell lines such as human liver cell line HepG2, renal proximal tubular cell lines (human HK-2 and porcine LLC-PK(1), and human epidermal keratinocyte cell line HaCaT. According to the findings, SiNPs were associated with a variety of harmful consequences, including lipid peroxidation, cell membrane disruption, mitochondrial dysfunction, apoptosis, and antiproliferative activity [13,14,15].

Notably and as previously mentioned, one of the most significant mechanisms of SiNP-induced toxicity is oxidative stress. The latter was described by Sies [16] as “a balance favoring oxidants over antioxidants, potentially causing damage”. The increased production of ROS has been linked to numerous diseases [17,18] as it can result in oxidative stress to molecules, cells, and tissues [19]. A rise in the concentration of oxidative damage indicators can indicate oxidative damage [20]. Reactive oxygen species are regulated by many antioxidant systems, including (1) low molecular antioxidants (alpha-tocopherol, ascorbic acid, GSH, and beta-carotene), and (2) cellular antioxidant enzyme systems such as superoxide dismutases (SOD), catalase, and glutathione peroxidase (GPx) [21,22,23]. Because glutathione (GSH) is a substrate for GPx, GPx becomes inactive when glutathione levels fall [24].

Antioxidants are utilized to decrease oxidative stress, and among them is magnesium (Mg). Magnesium has a high antioxidant activity in restoring oxidative damage by directly affecting metabolic and physiologic processes. Magnesium is necessary for optimal metabolic activities such as biomolecule synthesis and stability, as well as mitochondrial function [25]. It is a cofactor of the enzymes involved in the biosynthesis of the essential cellular antioxidant, GSH, responsible for maintaining the cell’s redox status [26]. Mg deficiency can be associated with oxidative damage and lipid peroxidation, and it reduces GSH activity, which may cause endothelial dysfunction [27].

It is well-recognized that magnesium protects against oxidative damage and lipid peroxidation. It has been proposed that Mg, by enhancing the concentrations of reduced GSH and the activity of SOD, decreases the free-radical-mediated peroxidative damage [28]. Possible mechanisms for magnesium’s anti-oxidative activity include inhibiting lipid peroxidation and promoting the levels of restored glutathione in cells [29]. Mg concentration and GSH levels in human blood are positively correlated [30]. Cell death and free radical formation are both increased when the GSH concentrations are low [31]. Hence, the current study was conducted to assess the toxic effects of oral SiNPs on the kidneys, liver, and adrenal glands in rats and to study the potential role of magnesium co-supplementation.

## 2. Materials and Methods

### 2.1. Chemicals

SiNPs were purchased from Sigma-Aldrich Chemical Co. (St. Louis, MO, USA) of the catalog number 637246 and 5–20 nm particle size (TEM). The SiNPs were white in color and powdered in appearance. The specification description labeled by the manufacturer of the SiNPs utilized in our study is shown in Table 1. Magnesium sulfate (MgSO_4_) was also purchased from Sigma-Aldrich.

### 2.2. Preparation of SiNPs Suspension

The solution of SiNPs was prepared by sonication according to the method of Canesi et al. [32]. Briefly, SiNPs were dispersed in distilled water, followed by sonication for 15 min at 100 W (Vibra-Cell sonicator, Sonics, Newtown, CN, USA) in an ice-water bath. The size, the polydispersity index (PI), and the Zeta Potential of the SiNPs in the solution were determined using a dynamic light scattering analyzer (ZETASIZER ULTRA, Malvern Instruments, Malvern, Worcestershire, UK).

### 2.3. Animals and Experimental Design

A total of 24 Sprague Dawley male rats weighing 0.2 kg were obtained from the Medical Experimental Research Center (MERC) (Mansoura University, Egypt). They were kept in ventilated animal rooms in polypropylene cages. They were housed in the following settings: 22–25 °C, a humidity range of 60–10%, and regular 12-h daylight. All of the experimental animals in this study were handled following the Guide for the Care and Use of Laboratory Animals [33]. Rats were divided into four groups (six rats each) as follows:

Group I (control group): Rats administered distilled water daily for 90 days.

Group II: Rats received Mg supplementation at a dose of 50 mg/kg/d by oral gavage for 90 days [28].

Group III: Rats received SiNPs dissolved in distilled water at a dose of 100 mg/kg/d by oral gavage for 90 days [34].

Group IV: Rats received SiNPs at a dose the same as in group III and Mg supplementation at a dose the same as in group III.

All of the rats were observed for 90 days. At the end of the study, all of the rats were sacrificed under anesthesia. Three milliliters of blood were collected from the abdominal aorta under anesthesia for the estimation of the liver transaminases, serum creatinine, and cortisol values. The liver, kidneys, and adrenal gland of the sacrificed rats were then excised and weighed, and then divided into several pieces for the evaluation of the oxidant-antioxidant status and histological changes in each of them.

### 2.4. Quantitative Estimation of Liver Enzymes, Serum Creatinine, and Cortisol Levels

According to the manufacturer’s prescriptions, the activity of the hepatic biomarkers (ALT and AST) and level of creatinine as a marker of renal tissue injury were estimated in the serum by the ready-made colorimetric kits purchased from Spinreact^®^ (Esteve De Bas, Spain). The serum cortisol level was estimated as per the manufacturer’s protocol of the commercially available validated ELISA kits (Cat #MBS727040, Mybiosource Inc.^®^, San Diego, CA, USA), which possess an acceptable (10%) inter- and intra-assay CV%.

### 2.5. Assessment of Oxidative Stress Markers

Nine milliliters of phosphate-buffered saline were used to homogenize 1 g (1:10 *w*/*v*) of each excised organ (liver, kidney, and adrenal). To remove cell debris, homogenates were centrifuged at 750× *g* for 10 min at 4 °C. After this, the concentration of reduced glutathione (GSH) as an antioxidant biomarker and malondialdehyde (MDA) as an oxidant parameter were spectrophotometrically estimated as mmol/g wet tissue in the supernatant of centrifuged tissue extract [35], by using the validated kits provided by Bio-diagnostic^®^ (Giza, Egypt) according to the enclosed pamphlets.

### 2.6. Histopathological Examination

The liver, kidneys, and adrenal glands were surgically removed from the animals. Slices were obtained and the blood was removed by washing in ice-cold normal saline (0.9% NaCl) and 20 mM EDTA. They were immediately divided into small pieces and preserved for 48 h in 10% phosphate-buffered formalin. After that, the tissues were put in 70% ethyl alcohol and kept at −20 °C until processing. For histological inspection, the tissue specimens were treated, embedded in paraffin, sectioned at 0.1 mm, and stained with hematoxylin and eosin (H&E) [2]. Photomicrographs were taken by B-193 optical microscope (Optika Microscope, Ponteranica, Italy) equipped with a 5 megapixel C-B5 digital camera (Optika, Italy).

### 2.7. Statistical Analysis

Data were analyzed by IBM SPSS Corp., Released in 2013., IBM SPSS Statistics for Windows, V22.0. Armonk, NY, USA: IBM Corp. Data were presented as means and standard deviations for parametric data following testing normality using the Kolmogorov–Smirnov test. The significance of the result was set at a 0.05 level. One-way ANOVA test was used for comparison between more than two independent groups.

## 3. Results

### 3.1. Characterization of SiNPs

**Figure 1:**
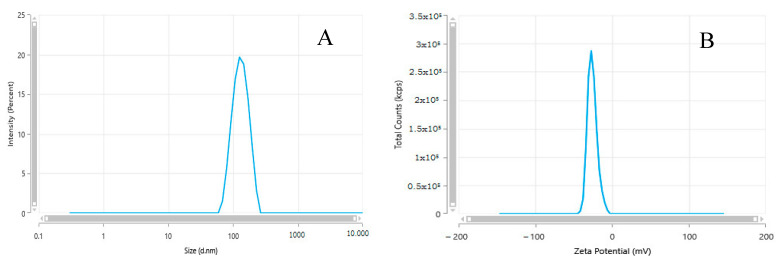
Representative hydrodynamic size (**A**) and Zeta potential (**B**) of the SiNP suspension.

The dynamic light scattering was utilized to study SiNPs’ stability (against coalescence) in solution. SiNPs tended to aggregate/agglomerate in distilled water, with a mean hydrodynamic size in solution of 160.4 ± 3.17 nm (Figure 1A). The mean measured zeta potential of SiNPs was −25 ± 0.85 mV (Figure 1B) and this could have reduced the electrostatic repulsion of the particles, leading to some aggregation. Zeta potential values more positive than +30 mV and more negative than −30 mV are generally considered to have good stability against coalescence [36]. The mean polydispersity index (PI) of the SiNPs was 0.2374 ± 0.03.

### 3.2. Clinical Signs and Mortality

All of the study animals underwent daily post-treatment checks for health, mortality, and any toxicity-related clinical signs. Neither clinical toxicities nor animal deaths occurred during the experimental period, after the oral administration of SiNPs.

### 3.3. Hepatic Function Assessment

The results of the liver enzymes assessed 90 days after the application of SiNPs are shown in Table 2. The SiNP group revealed a noticeable increase in both ALT and AST levels with high statistical significance (*p* < 0.001) when compared with the control rats. Moreover, the co-administration of Mg with SiNPs resulted in a significant reduction in ALT and AST levels (*p* = 0.001 and 0.009 respectively) in comparison with the SiNPs group. No statistical difference was detected between the Mg and control groups regarding the ALT and AST levels (*p* = 0.997 and 0.611, respectively).

### 3.4. Renal Function Assessment

As shown in Table 3, rats that received SiNPs showed a marked increase in creatinine levels with a high statistical significance in comparison with the control group (*p* < 0.001). The co-administration of Mg with SiNPs caused a marked decrease in creatinine levels with a high statistical significance in comparison with the SiNPs group (*p* < 0.001). No statistical difference was detected between the Mg and control groups regarding creatinine levels (*p* = 0.239).

### 3.5. Adrenal Function Assessment

Table 4 shows that the cortisol levels in the SiNPs group revealed a marked increase compared with the control rats with a high statistical difference (*p* < 0.001). Co-administration of Mg with SiNPs resulted in a marked decrease in cortisol values with a statistical significance versus the SiNPs group (*p* = 0.006). No statistical difference was found between Mg and control groups as regards cortisol levels (*p* = 0.385).

### 3.6. Histopathological Examination

**Figure 2:**
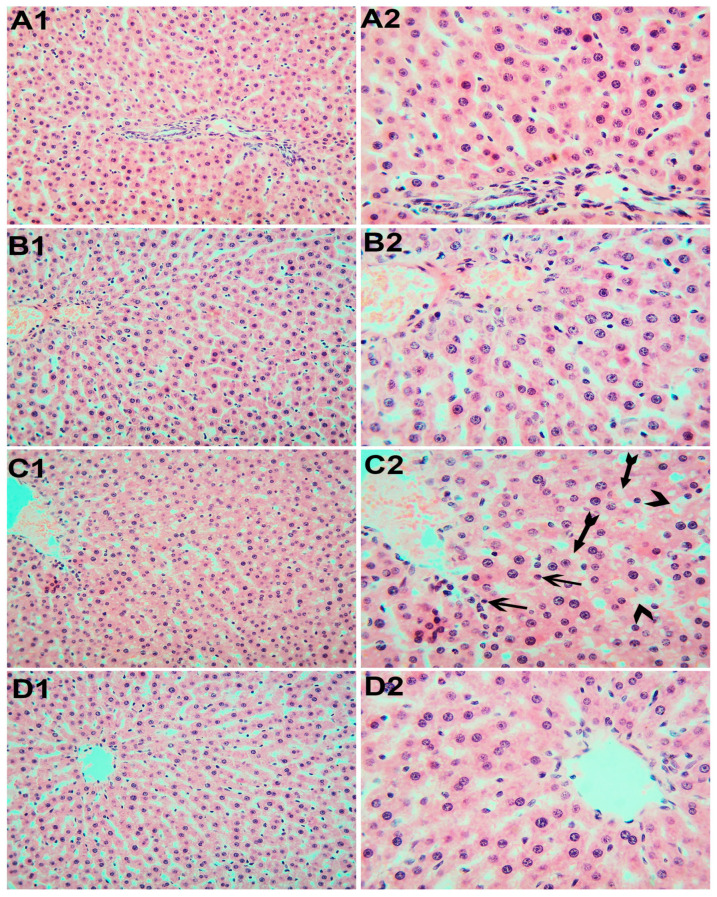
Representative liver sections photomicrographs. (**A1**,**A2**) Liver of the control rats. (**B1**,**B2**) Rats that received Mg. (**C1**,**C2**) Rats exposed to SiNPs showing sinusoidal dilatation (arrowheads). The hepatocytes’ nuclei are of variable size with pyknosis of some nuclei (arrows). Most of the hepatocytes show cytoplasmic vacuolation (tailed arrows). (**D1**,**D2**) Rats exposed to SiNPs and received Mg, showing no obvious changes in the hepatocytes and hepatic sinusoids. H&E. (**A1**,**B1**,**C1**,**D1**) ×20 magnification. (**A2**,**B2**,**C2**,**D2**) ×40 magnification.

Liver sections of the control rats (Figure 2A1,A2) and those that received Mg (Figure 2B1,B2) were extremely similar and revealed a normal structure and arrangement of the periportal islands of well-preserved hepatocytes interspersed with normal sinusoidal structures. However, hepatic tissue from the rats exposed to SiNPs (Figure 2C1,C2) showed a widening of the hepatic sinusoids and vacuolized hepatocytes with varying degrees of contracted (pyknotic) nuclei. Rats exposed to SiNPs and who received Mg (Figure 2D1,D2) showed healthy liver tissue and most hepatocytes appeared to be polygonal with a clear acidophilic cytoplasm and vesicular nuclei.

**Figure 3:**
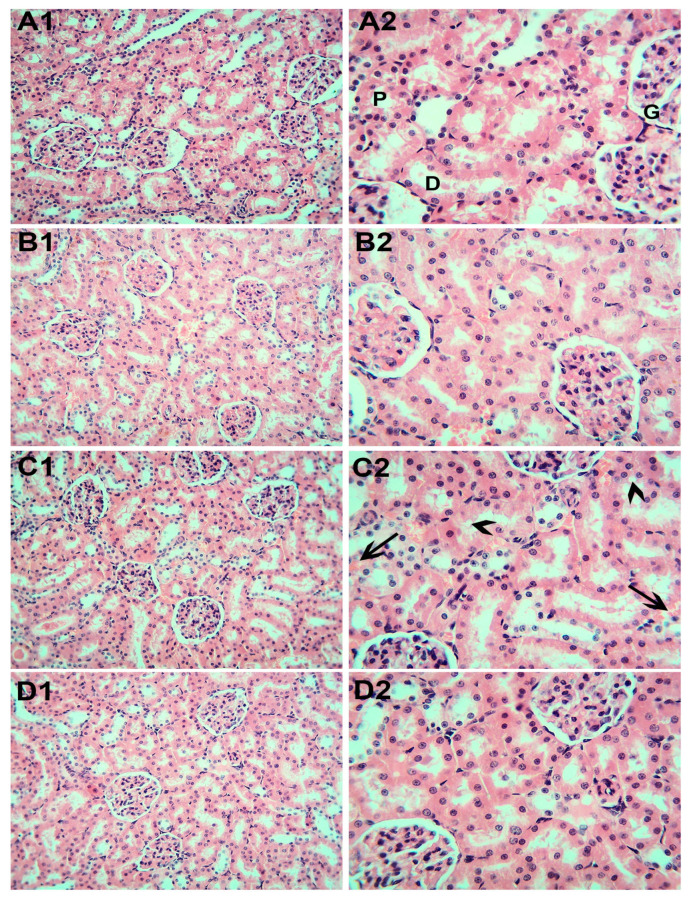
Representative kidney section photomicrographs. (**A1**,**A2**) Control rats demonstrating the normal structure of the glomeruli (G), proximal (P), and distal (D) convoluted tubules. (**B1**,**B2**) Rata that received Mg. (**C1**,**C2**) Rats exposed to SiNPs showing inter-tubular congestion (arrows) with swelling and vacuolation of the endothelial lining of the proximal and distal convoluted tubules (arrow heads). (**D1**,**D2**) Rats exposed to SiNPs and that received Mg showing normal cellularity of the glomerulus and renal tubules. H&E. (**A1**,**B1**,**C1**,**D1**) ×20 magnification. (**A2**,**B2**,**C2**,**D2**) ×40 magnification.

As shown in Figure 3, the kidney specimens from the control rats (A1,2) and those that received Mg (B1,2) showed normal histological architecture of the renal cortex with the glomeruli formed by a tuft of capillaries and surrounded by the Bowman’s space, the proximal convoluted tubules with a narrow cavity and cuboidal endothelial lining, and distal convoluted ones with wider lumens and more flattened lining. C1 and 2 is for rats exposed to SiNPs, which show inter-tubular congestion and pronounced tubular degenerative changes. Regarding the kidney sections from the rats exposed to SiNPs and that received Mg (D1,2), there were indetectable alternations in the glomeruli and epithelial cells lining the tubules in the cortical part.

**Figure 4:**
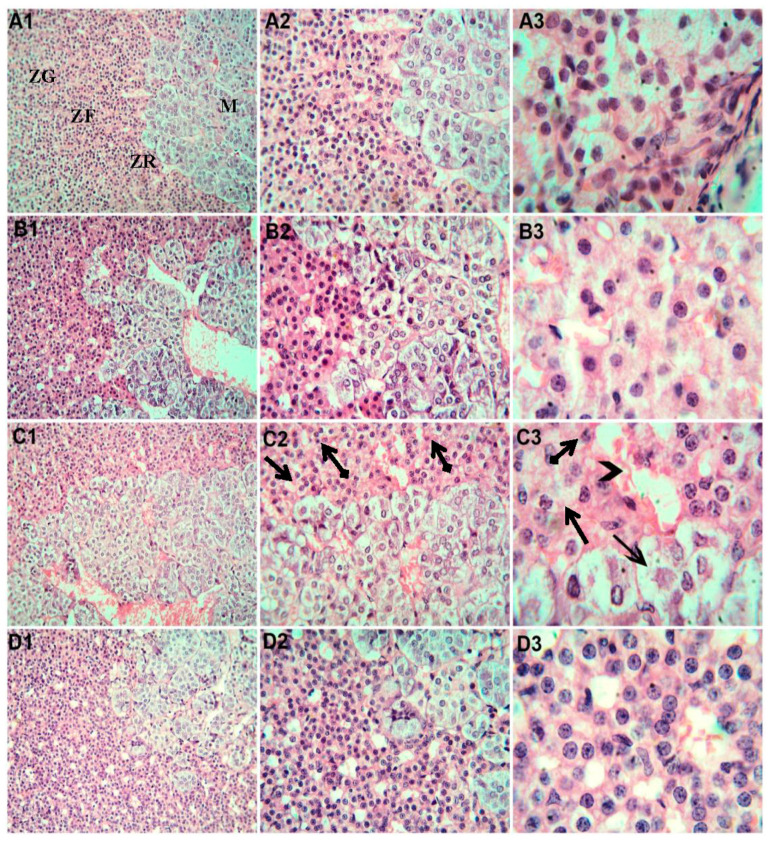
Representative adrenal gland sections photomicrographs. (**A1**,**A2**,**A3**) Control rats demonstrating the normal structure of the zona glomerulosa (ZG), zona fasciculata (ZF), zona reticularis (ZR), and medulla (M). (**B1**,**B2**,**B3**). Rats that received Mg and showing normal histological architecture and arrangement of cells within the adrenal cortex and medulla. (**C1**,**C2**,**C3**) Rats exposed to SiNPs showing disorganized cell cords interspersed with distended blood sinusoids (arrow heads) and abnormal cortical cells, which show vacuolated cytoplasm (arrows) and pyknotic nuclei (tailed arrows). H&E. (**A1**,**B1**,**C1**,**D1**) ×20 magnification. (**A2**,**B2**,**C2**,**D2**) ×40 magnification. (**A3**,**B3**,**C3**,**D3**) ×100 magnification.

As illustrated in Figure 4, the adrenal gland specimens from the control rats (A1,2,3) and that received Mg (B1,2,3) showed normal histological architecture of the adrenal medulla and cortex that formed from polyhedral cells with rounded vesicular nuclei separated by vascular sinusoids within normal limits and arranged as rounded clusters in the zona glomerulosa and long straight cords in the zona fasciculata. Zona fasciculata and reticularis in the rats exposed to SiNPs (C1,2,3) showed a disturbed architecture and dilated blood sinusoids. Most cortical cells appeared swollen and vacuolated, while the others appeared contracted (shrunken) with the pyknotic nuclei. Rats exposed to SiNPs and that received Mg (D1,2,3) showed histological changes through amelioration and an almost normal adrenal cortex.

### 3.7. Oxidative Stress Assessment

The tissue levels of GSH and MDA mmol/g in the wet tissue are shown in Table 5. In the liver, kidneys, and adrenal glands, the group that received SiNPs showed significant reductions in GSH levels and significant increases in MDA levels (*p* < 0.001) compared with the control group. The co-administration of Mg with SiNPs resulted in marked increases in GSH and decreases in MDA levels with a high statistical significance compared with the SiNPs group (*p* < 0.001). There was no statistical difference in GSH and MDA levels between the Mg and control groups.

### 3.8. Weight of the Liver, Kidneys, and Adrenal Glands

As shown in Table 6, there was no statistically significant difference in the liver weight in the SiNPs group compared to the control (*p* = 0.066). However, compared with those in the control group, there was a significant increase in the kidney (*p* < 0.001) and adrenal gland weight (*p* < 0.001).

For the SiNPs + Mg group, although the liver and kidney weight were decreased in comparison with the SiNPs group, no statistically significant differences were reported (*p* = 0.513 and 0.151, respectively). On the other hand, there was a statistically significant decrease in the adrenal weight in comparison with the SiNPs group (*p* = 0.021).

## 4. Discussion

Silica nanoparticles are extensively used in a variety of consumer products, and so human exposure seems inevitable [7]. The toxic effects of SiNPs are related to their small size and its shape [9]. SiNPs have been reported to have toxic effects on the kidneys, liver, lungs, and spleen in rats [37]. In addition, they increase the synthesis of ROS with the subsequent induction of oxidative stress [12].

The aim of the current study was to investigate whether repeated the (subchronic) oral administration of silica nanoparticles for 90 days could have harmful effects on the liver, kidneys, and adrenal glands of Sprague-Dawley rats, as well as the potential protective role of magnesium. There were no fatalities or treatment-related clinical toxicity symptoms seen in the rats throughout the experimental period. In our study, we administered SiNPs via the gavage, as the oral administration of SiNPs exhibited higher rates of absorption and distribution than those administered via other routes of administration in the majority of in vivo toxicity studies [11].

According to Lee and co-workers [38], the kidneys, liver, lungs, and spleen were the target organs of SiNPs (20 nm and 100 nm) given orally to rats. The liver is more vulnerable than other organs because it is the main organ engaged in human metabolism and detoxification.

Based on our findings, oral exposure to SiNPs resulted in a considerable rise in ALT and AST levels when compared with the control group, suggesting that these particles can harm the liver. Furthermore, the histology of liver sections showed some changes, including widening of the hepatic sinusoids, some degree of vacuolated cytoplasm and pyknosis of some hepatocyte nuclei in the SiNP-treated group, which further supported hepatic damage.

Such findings are in agreement with the research done by van der Zande et al. [39], using SiNPs (2500 mg/kg bw/d for 84 days orally) that revealed an increased incidence of hepatic fibrosis in rats. Furthermore, according to Liu et al. [40], intraperitoneal injection of 110-nm mesoporous hollow silica NPs in mice (at 50 mg/kg twice per week for 6 weeks) can significantly raise the level of the aminotransferase (AST) and cause marked histopathological changes in the liver. Mehdi and Al-Husseini [34] observed that serum ALT and AST values saw a considerable increase in the rats orally exposed to 150 mg/kg SiNPs for 30 days compared with the control group, with no significant difference in the group given 100 mg/kg SiNPs for 30 days.

Moreover, Liu and co-workers [41] reported that SiNPs accumulated mainly in mononuclear phagocytic cells in mice livers and can induce hepatotoxicity in the form of a rise in the serum ALT and AST levels and the lymphocytic infiltration, micro granulation, and degenerative necrosis of hepatocytes. Hassankhani and colleagues [11] reported that mice subjected to SiNPs (333.33 mg/kg/d) for 5 days showed congestion, hepatocyte oedema, and cellular necrosis.

In contrast with our results, Kim et al., in 2014 [2], reported no histopathological changes, nor an increase in the liver enzymes in rats given SiNPs 500 mg/kg orally for 90 days. This might be explained by the differences in the study design, dose, and SiNPs size.

Indeed, as the kidneys filter waste from the blood, some NPs should be eliminated by them. SiNPs can be found in renal tissue and can lead to abrupt biochemical and pathologic alterations [42,43].

Our results found that direct exposure to SiNPs caused nephrotoxicity, as evidenced by increased creatinine levels, increased kidney weight in the rats exposed to SiNPs, and the histopathological alterations in the kidneys in the form of swelling and vacuolation of the epithelial lining of the proximal and distal convoluted tubules.

Our results are concordant with Hassankhani et al. [11], who found that all of the mice subjected to SiNPs for 5 days had kidney damage, including hazy swelling, hydropic degeneration, necrosis of epithelial cells in renal tubules, congestion, distention of Bowman’s capsule, hyaline casts, glomeruli segmentation, and tubular swelling. Additionally, they reported that serum urea levels increased significantly, but were have been no obvious differences between the SiNPs-treated and control groups’ creatinine.

In general, and regarding the toxicity of oral SiNPs on the kidney, several earlier researches works have reported controversial findings. Nishimori et al. [44] reported that kidneys showed no histopathological changes in mice intravenously injected with 70-nm SiNPs for one month. Furthermore, van der Zande et al. [39] found that the kidneys demonstrated no differences between SiNPs-treated rats (2500 mg/kg/d for 84 days orally) and the controls in the histopathological assessment.

Additionally, our findings are in contrast with Kim et al. [45], who revealed that the liver and kidney weight for all the orally treated SiNPs groups (100 and 1000 mg/kg/d) were not significantly different compared with those of the control group over 12 weeks. No abnormal histopathological or biochemical alterations were noticed in the SiNPs group.

The results of earlier research, which revealed that the nanotoxicity of SiNPs was depending on their size and dose, may help explain the inconsistent findings regarding the toxic effects of oral SiNPs [46].

The current results suggested that SiNPs could have a toxic effect on the adrenal glands. There was a marked increase in the level of cortisol and the adrenal weight in the SiNPs group compared with the control. Moreover, histopathological examination revealed disturbed architecture of the adrenal cortex and dilated blood sinusoids. Most cortical cells appeared to have vacuolated cytoplasm and shrunken pyknotic nuclei. Previous studies supported this finding. For example, Almanaa et al. [47] reported a noticeably increased cortisol level in male rats given SiNPs orally (125 mg/kg/d) for 4 days.

The cortisol hormone may play a part in the risk of cardiovascular disease [48]. For example, enhanced arterial stiffness and compromised endothelial function have both been associated with elevated cortisol levels in those with Cushing’s disease [49]. Ducat et al. [50] and Koe et al. [51] reported that the increased release of the cortisol hormone made mice’s symptoms of anxiety worse.

In our study, magnesium supplementation caused a significant decrease in cortisol levels. It is controversial how magnesium lowers circulating cortisol levels, although modifications to the hypothalamic–pituitary–adrenal axis may be the mechanism through which this occurs [52]. Magnesium has been demonstrated to reduce the HPAA’s activity, including central ACTH and peripheral cortisol levels [53]. A high Mg content reduces the baseline synthesis of the inflammatory cytokines (TNF-a, IL-1, and IL-6), disrupting the expected level of cortisol [54].

In our study, SiNPs administration resulted in a marked reduction in GSH concentrations and a marked increase in MDA concentrations in the liver, kidneys, and adrenal tissues, indicating oxidative stress induction. Moreover, Mg administration together with SiNPs inhibited SiNP-induced ROS production, as our results showed increasing GSH levels and decreasing MDA levels in the liver, kidneys, and adrenal tissues.

Our data are in line with Hu et al., 2019 [35], who revealed that SOD and GSH levels in mice sera and liver significantly decreased and the levels of MDA, a product of lipid peroxidation, were significantly increased starting in week eight after the oral administration of SiNPs (200 mg/kg). They suggested that SiNPs increased ROS production through ER stress, which affected the nuclear-related factor 2 (Nrf2) pathway, resulting in enhanced ROS generation in mice.

Earlier studies have shown that SiNPs increase the production of ROS, which deplete endogenous antioxidants such as SOD and GSH and damages biologic macromolecules such as nucleic acids, lipids, and proteins through lipid peroxidation [55,56]. Lipid oxidation produces reactive lipid radicals including MDA and 4-hydroxynonenal (4-HNE), which cause DNA damage. The latter causes cell cycle arrest to allow for DNA repair and proteostasis, but if oxidative stress continues, cell death via apoptosis is encouraged [57].

In rats exposed to SiNPs, there was a significant increase in kidney and adrenal weights, but no significant increase in liver weight was observed. In the study by Liu et al., 2012 [40], no remarkable changes were detected in the liver and kidney weight (compared with the control group) after the intraperitoneal injection of SiNPs (50 mg/kg) in mice for 6 weeks.

The current results showed that the oral administration of magnesium (50 mg/kg/d) in rats given SiNPs orally (SiNPs + Mg) may have a protective effect against SiNPs toxicity. This was determined by the significant reduction in liver enzymes, creatinine, and cortisol; reduction in MDA; and increase in GSH, as well as the absence of histopathologic changes in the liver, kidneys, and adrenals in the group exposed to SiNPs. To the best of our knowledge, this work is the first to study the sub chronic effect of SiNPs on adrenal glands and to investigate the ameliorating effect of Mg on SiNP-induced toxicities.

Many functions, including antioxidant and anti-inflammatory responses, are regulated by Mg. It promotes mitochondrial activity and raises the GSH concentration, lowering oxidative stress. As a result, Mg may be an effective strategy to reduce inflammation and oxidative stress [58]. Mg acts as an indirect antioxidant because it is a cofactor of the enzymes that synthesize GSH, reducing the effects of oxidative stress on cell membrane stability [59]. Treatment with Mg salts activates nuclear-related factor 2 (Nrf2), which is an important endogenous antioxidant pathway that protects against oxidative stress during oxidative stress [60] and regulates the gene transcription and protein expression of antioxidant enzymes including catalase (CAT), SOD, and glutathione peroxidase (GPx) [61].

## 5. Conclusions

In conclusion, SiNPs exhibit negative in vivo toxicological consequences. More specifically, the liver, kidneys, and adrenal glands are the target organs of these particles, according to the biochemical and pathological analyses. SiNPs induced organ toxicities through the generation of ROS, GSH depletion, and MDA accumulation. Our findings indicat that magnesium may have an important role in mitigating the toxicological effects of SiNPs on the liver, kidneys, and adrenal glands. As a result, additional analyses of the link between toxicity and particle sizes, shapes, or chemical surface modification are required. Further studies on the mechanisms through which SiNPs induced oxidative stress are crucial.

## Figures and Tables

**Table 1:** Specifications of the SiNPs used in the current study.

Appearance (Form)	Surface Area	pH	Solid Content	Particle Size (nm)	Density	Bulk Density	Solvent	Purity
Nanopowder (spherical, porous)	590–690 m^2^/g (TEM)	3.7–4.7	24.0–30.0%	5–20 nm (TEM)	2.2–2.6 g/mL at 25 °C	0.068 g/mL	water	99.5% (Based on Trace Metals Analysis)

**Table 2:** Comparison between the studied groups regarding the serum alanine aminotransferase and aspartate aminotransferase levels in the studied groups.

Parameter	Study Groups				ANOVA Test *p*-Value *
	Control Group (N = 6)	Mg Group (N = 6)	SiNPs Group (N = 6)	SiNPs + Mg Group (N = 6)	
ALT (U/L)	24 ± 4.47	23.50 ± 5.01 ^bc^	47.50 ± 3.94 ^a^	35 ± 4.52 ^ab^	F = 37.975 *p* < 0.001 **
AST (U/L)	100.83 ± 11.02	94.50 ± 5.39 ^bc^	152 ± 11.83 ^a^	133.50 ± 4.85 ^ab^	F = 56.66 *p* < 0.001 **

The data are expressed as mean ± SD. SD: standard deviation. SiNPs: silica nanoparticles. Mg: magnesium. ALT: alanine aminotransferase. AST: aspartate aminotransferase. N: number. U/L: units per liter. *: Significant (*p* ˂ 0.05). **: Highly significant (*p* ≤ 0.001). ^a^: Significant in relation to the control group. ^b^: Significant in relation to SiNPs group. ^c^: Significant in relation to SiNPs + Mg group.

**Table 3:** Comparison between the studied groups regarding the serum creatinine levels.

	Study Groups				ANOVA Test *p*-Value *
	Control Group (N = 6)	Mg Group (N = 6)	SiNPs Group (N = 6)	SiNPs + Mg Group (N = 6)	
Mean ± SD (mg/dL)	0.56 ± 0.06	0.41 ± 0.08 ^bc^	1.79 ± 0.20 ^a^	0.95 ± 0.15 ^ab^	F = 126.730 *p* < 0.001 ** p < 0.001 **

SD: standard deviation. SiNPs: silica nanoparticles. Mg: Magnesium. N: number. mg/dL: milligrams per deciliter. *: significant (*p* ˂ 0.05). **: Highly significant (*p* ≤ 0.001). ^a^: significant in relation to the control group. ^b^: significant in relation to SiNPs group. ^c^: significant in relation to SiNPs + Mg group.

**Table 4:** Comparison between the studied groups as regards serum cortisol in the studied groups.

		Study Groups			ANOVA Test *p*-Value *
	Control Group (N = 6)	Mg Group (N = 6)	SiNPs Group (N = 6)	SiNPs + Mg Group (N = 6)	
Mean ± SD (µg/dL)	0.25 ± 0.04	0.22 ± 0.02 ^bc^	0.37 ± 0.04 ^a^	0.30 ± 0.02 ^ab^	F = 28.398 *p* < 0.001 **

SD: standard deviation. SiNPs: silica nanoparticles. Mg: magnesium. N: number. µg/dL: micrograms per deciliter. *: significant (*p* ˂ 0.05). **: highly significant (*p* ≤ 0.001). ^a^: significant in relation to the control group. ^b^: significant in relation to the SiNPs group. ^c^: significant in relation to SiNPs + Mg group.

**Table 5:** Comparison between the studied groups regarding tissue levels of GSH and MDA (mmol/g wet tissue) in the studied groups.

Parameters			Study Groups			ANOVA Test *p*-Value *
		Control Group (N = 6)	Mg Group (N = 6)	SiNPs Group (N = 6)	SiNPs + Mg Group (N = 6)	
Liver	GSH (mean ± SD)	2.72 ± 0.12	2.90 ± 0.07 ^bc^	1.38 ± 0.19 ^a^	1.97 ± 0.09 ^ab^	F = 188.405 *p* < 0.001 **
	MDA (mean ± SD)	5.89 ± 0.37	4.92 ± 0.54 ^bc^	14.80 ± 1.11 ^a^	10.85 ± 1.07 ^ab^	F = 182.136 *p* < 0.001 **
Kidney	GSH (mean ± SD)	1.58 ± 0.15	1.69 ± 0.11 ^bc^	0.81 ± 0.11 ^a^	1.18 ± 0.04 ^ab^	F = 81.313 *p* <0.001 **
	MDA (mean ± SD)	4.76 ± 0.26	4.02 ± 0.14 ^bc^	11.13 ± 0.98 ^a^	7.59 ± 0.85 ^ab^	F = 141.260 *p* < 0.001 **
Adrenal	GSH (mean ± SD)	1.47 ± 0.10	1.55 ± 0.05 ^bc^	0.70 ± 0.09 ^a^	8 ± 0.46 ^a^	F = 127.623 *p* < 0.001 **
	MDA (mean ± SD)	3.92 ± 0.30	3.53 ± 0.25 ^bc^	1.09 ± 0.09 ^ab^	6.22 ± 0.16 ^ab^	F = 269.944 *p* < 0.001 **

SD: standard deviation. SiNPs: silica nanoparticles. Mg: magnesium, GSH: glutathione. MDA: malondialdehyde. N: number. *: significant (*p* ˂ 0.05). **: highly significant (*p* ≤ 0.001). ^a^: significant in relation to the control group. ^b^: significant in relation to SiNPs group. ^c^: significant in relation to SiNPs + Mg group.

**Table 6:** Comparison between the studied groups regarding the weight of theliver, kidneys, and adrenal glands in the studied groups.

Parameters	Study Groups				ANOVA Test *p*-Value *
	Control Group (N = 6)	Mg Group (N = 6)	SiNPs Group (N = 6)	SiNPs + Mg Group (N = 6)	
Liver weight (g)	5.43 ± 1.14	4.81 ± 0.14 ^bc^	6.36 ± 0.23	5.87 ± 0.33	F = 7.085 *p* = 0.002 *
Kidney weight (g)	1.19 ± 0.11	1.05 ± 0.05 ^abc^	1.42 ± 0.09 ^a^	1.32 ± 0.04 ^a^	F = 25.510 *p* < 0.001 **
Adrenal weight (g)	0.06 ± 0.01	0.05 ± 0.01 ^bc^	0.08 ± 0.01 ^a^	0.07 ± 0.01 ^ab^	F = 31 *p* < 0.001 **

The data are presented as mean ± SD. SiNPs: silica nanoparticles. Mg: magnesium, SD: standard deviation. *: Significant (*p* ˂ 0.05). **: highly significant (*p* ≤ 0.001). ^a^: significant in relation to the control group. ^b^: significant in relation to SiNPs group. ^c^: significant in relation to SiNPs + Mg group.

## Data Availability

All data are contained within the article.
